# Therapeutic Effects and Molecular Mechanism of Chlorogenic Acid on Polycystic Ovarian Syndrome: Role of HIF-1alpha

**DOI:** 10.3390/nu15132833

**Published:** 2023-06-21

**Authors:** Zhenghong Zhang, Congjian Shi, Zhengchao Wang

**Affiliations:** Provincial Key Laboratory for Developmental Biology and Neurosciences, College of Life Sciences, Fujian Normal University, Fuzhou 350007, China; zhangzh@fjnu.edu.cn (Z.Z.); scongjian@163.com (C.S.)

**Keywords:** chlorogenic acid, hypoxia inducible factor-1alpha, follicular development, hormone synthesis, inflammatory response, oxidative stress, polycystic ovarian syndrome

## Abstract

Chlorogenic acid (CGA) is a powerful antioxidant polyphenol molecule found in many diets and liquid beverages, playing a preventive and therapeutic role in various diseases caused by oxidative stress and inflammation. Recent research has found that CGA can not only improve clinical symptoms in PCOS patients but also improve follicular development, hormone status, and oxidative stress in PCOS rats, indicating the therapeutic effect of CGA on PCOS. Notably, our previous series of studies has demonstrated the expression changes and regulatory mechanisms of HIF-1alpha signaling in PCOS ovaries. Considering the regulatory effect of CGA on the HIF-1alpha pathway, the present article systematically elucidates the therapeutic role and molecular mechanisms of HIF-1alpha signaling during the treatment of PCOS by CGA, including follicular development, steroid synthesis, inflammatory response, oxidative stress, and insulin resistance, in order to further understand the mechanisms of CGA effects in different types of diseases and to provide a theoretical basis for further promoting CGA-rich diets and beverages simultaneously.

## 1. Introduction

Polycystic ovary syndrome (PCOS) is the most common endocrine disease, with female infertility, anovulation, and hyperandrogenism, which even affects the quality of life after menopause [1,2,3,4,5]. The main clinical symptoms include hormonal imbalances, irregular menstrual cycles, dysfunction of follicle maturation, and miscarriage [6,7,8]. It is worth noting that the systemic low-grade inflammation in PCOS patients is closely related to oxidative stress [9,10,11]. While oxidative stress is typically present in PCOS patients, leading to an increase in the number of lipid peroxidation products and other highly toxic products such as malondialdehyde [8], it also plays an important role in infertility [12,13,14]. Therefore, alternative treatment strategies to reduce oxidative stress can improve the reserve and developmental ability of ovarian follicles in PCOS patients [15,16,17,18].

At present, in-depth research has been conducted on PCOS pathogenesis and treatment strategies. We have clearly demonstrated that the hypoxia-inducible factor (HIF)-1alpha signaling pathway plays an important regulatory role during this process [19,20,21,22,23,24]. HIF-1alpha is a regulatory alpha-subunit of heterodimeric transcription factor HIF-1, mainly expressed in granulosa cells, regulated by the hypothalamus-pituitary-gonad axis, and involved in follicular development, ovarian ovulation, and hormone synthesis [20,21,24,25,26]. HIF-1alpha is indispensable in the dimethyldiguanide (DMBG) treatment of PCOS as a novel therapeutic target [20,27,28,29].

In recent years, the importance of food-based alternative drugs and free drugs in the treatment of PCOS has received widespread attention. Phytochemical substances in medicinal plants are the source of effective treatment for diseases such as PCOS [30,31,32,33,34,35,36,37]. They can improve insulin sensitivity, promote ovulation, and reduce hyperandrogenism without side effects [30,32,34,38]. Among them, chlorogenic acid (CGA) is a widely distributed natural compound with many pharmacological activities, mainly extracted from natural plants such as honeysuckle, Eucommia ulmoides, coffee beans, and sunflowers [33,39,40,41]. It has been widely used in industries such as medicine, health, and food chemicals.

CGA is a polyphenol molecule with a strong antioxidant effect that is widely present in many diets and liquid drinks. It can eliminate free radicals in the body and plays a preventive and therapeutic role in many diseases, such as diabetes, hypertension (HPT), and atherosclerosis (AS) [39,40,42,43,44]. CGA not only has antioxidant activity but also has a series of biological functions such as liver anti-inflammatory protection and neuroprotection [39,40,41,42,43,44]. Recent studies have found that CGA also has therapeutic effects on PCOS [30,34,35,36,37]. However, the molecular regulatory mechanism of CGA in the treatment of PCOS still needs to be systematically elucidated.

Given the regulatory effect of CGA as an antioxidant on the HIF-1alpha signaling pathway, this article takes HIF-1alpha as a breakthrough point to systematically elucidate the role and molecular mechanisms of HIF-1alpha signaling during the treatment of PCOS with CGA, including follicular development and ovulation, steroid hormone synthesis, inflammatory response, oxidative stress, and insulin resistance.

## 2. Overviews of Chlorogenic Acid (CGA)

CGA is widely distributed in the plant kingdom as coffee tannic acid, ranging from dicotyledonous plants to ferns, and is mainly present in Lonicera and Artemisia plants [44]. A high content of CGA exists in plants such as Eucommia ulmoides, honeysuckle, coffee, and chrysanthemum [45]. In addition, vegetables and fruits also contain CGA, such as potatoes, carrots, spinach, and apples [44,45,46].

The chemical name of CGA is 3-O-caffeoylquinic acid, C_16_H_18_O_9_, with a molecular weight of 354.30 [21,47]. Its semihydrate is a white or yellow needle shaped crystal that becomes an anhydrous compound at 110 °C, with a melting point of 206–208 °C [48]. At 25 °C, the solubility in water is relatively low, about 4% [48]. In hot water, solubility increases and changes with temperature. CGA is a polar organic acid that is unstable and prone to isomerization during the extraction process [44,47,48].

**Figure 1:**
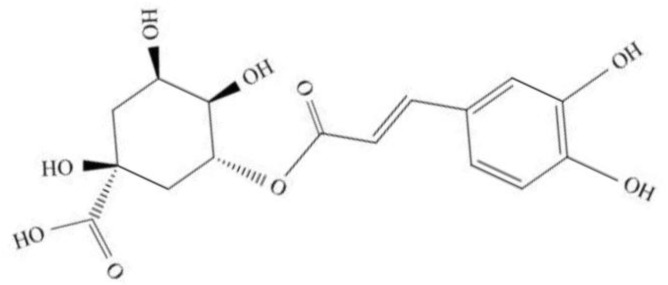
The molecular structure of chlorogenic acid (CGA).

The catechol hydroxyl contained in the molecular structure of chlorogenic acid is the most suitable reaction substrate for phenolase catalysis (Figure 1). It is easily oxidized under heat and light, which is also the key reason for the browning of many fruits containing CGA, such as peaches and apples [21,45]. Under alkaline conditions, CGA can undergo hydrolysis to form green quinones. CGA present in plants is often a mixture rather than a single component, including monocaffeioyl quinic acid, dicaffeioyl quinic acid, tricaffeioyl quinic acid, and methyl chlorogenic acid [44,47].

CGA is a kind of phenylpropanoids, which are effective phenolic antioxidants [1,44]. As we know, CGA can effectively eliminate free radicals, maintain normal functions, and also prevent disease occurrence [43,48]. For example, CGA can up-regulate the expressions of PPARα and SREBP-1, which are involved in liver lipid metabolism and restore diabetes and oleic acid-induced NAFLD [39,46]. Similarly, CGA can prevent protein glycosylation by regulating glycogen production and gluconeogenesis, thus participating in glucose metabolism [44].

Recently, some studies on CGA have found that CGA not only improves follicular development and oxidative stress in PCOS rats but also improves the inflammatory response in PCOS patients, indicating that CGA also has therapeutic effects on PCOS.

## 3. Polycystic Ovary Syndrome (PCOS)

**Figure 2:**
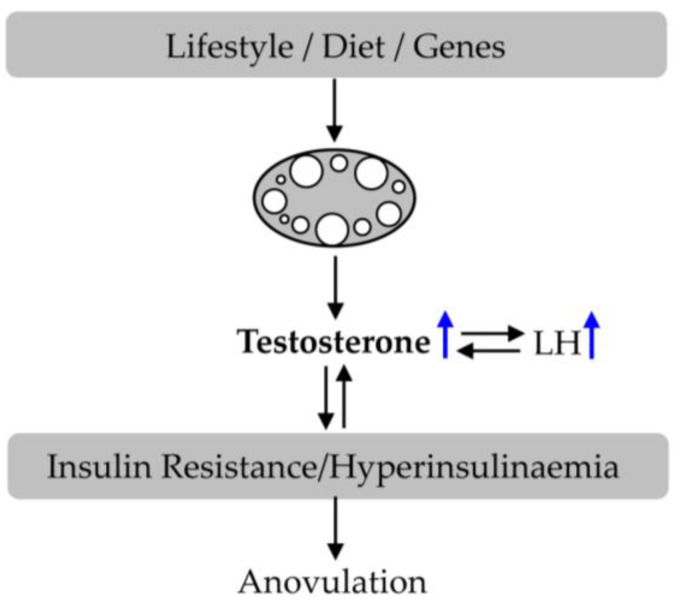
An increase in testosterone and LH leads to insulin resistance and anovulation in PCOS.

In 1935, Stein and Leventhal first described female PCOS [49], characterized by ovulatory dysfunction, hyperandrogenism, and polycystic ovary, accompanied by neuroendocrine features such as increased serum luteinizing hormone (LH) concentrations (Figure 2) [5,50,51,52,53,54]. Subsequently, a series of studies were conducted on the etiology, diagnosis, and treatment of PCOS [8,55,56,57].

The etiology of PCOS is very complex, mainly caused by genetic and environmental factors [6,8,58]. Unhealthy lifestyles, dietary habits, and infectious agents all increase the risk of its onset [58]. Due to insulin resistance and its elevated levels, ovarian function is disrupted, and androgen levels are elevated, leading to anovulation [8]. GnRH, FSH, LH, and prolactin levels in PCOS patients can also be disrupted [50,51,59]. The severity of PCOS increases with increasing levels of insulin and androgen. On the one hand, hyperinsulinemia can affect the synthesis and secretion of androgen levels in ovarian theca cells, reducing the biosynthesis of liver SHBG and IGFBP-1 [5,8,60]. On the other hand, an increase in androgen levels can stimulate visceral adipose tissue (VAT) and produce free fatty acids (FFA), leading to insulin resistance [5,8,54]. In addition, genetic predisposition, autoimmune disorders, and chronic inflammation are also important pathogenic factors for PCOS [61,62,63,64].

The diagnosis of PCOS is currently made according to the phenotypes of PCOS patients as described in the Rotterdam criteria (Table 1), which should be clearly indicated when diagnosing PCOS, including irregular menstrual cycles, elevated androgen levels, and exited cysts. The medical history and examination of suspected PCOS patients will be evaluated, while their hormone concentrations will also be tested to rule out similar diseases [65,66,67]. For example, in anovulatory patients, thyroid hormone is measured to exclude thyroid dysfunction, and prolactin is detected to exclude hyperprolactinemia [7,68]. Additionally, 17-hydroxyprogesterone is measured during the preovulation phase to confirm adrenal 21-hydroxylase deficiency or ovarian androgen excess [12,69].

The treatment of PCOS depends on the phenotype, focus, and goals of these patients. The purpose of PCOS treatment is to normalize the endometrium, counteract the effects of androgen, and reduce insulin resistance [12,17]. For example, androgen blockade is only related to hirsutism, while androgen inhibition is typically associated with acne [6]. In addition, for patients who do not pursue conception and are not contraindicated by hormone contraception, combined oral contraceptive therapy should be considered as part of the initial treatment [46]. Transdermal combination contraceptives or contraceptives containing only progesterone can be considered for patients intolerant to contraceptives, while slimming and fitness should be their first-line treatment for obese patients [46,65,66,67]. For PCOS patients with metabolic disorders, insulin sensitizers should also be considered, especially DMBG [28,29,62,70]. For patients who wish to have immediate fertility, oral ovulation agents should be considered [71]. PCOS treatment usually means lifelong follow-up and multiple treatments, including various treatment methods, depending on the patient’s performance, complications, wishes, and goals [15,16,17,26,72,73].

Finally, although PCOS is a heterogeneous disease that is not caused by a single factor, a deeper understanding of the underlying mechanisms behind ovarian pathophysiological changes in PCOS patients can help us develop effective treatment methods to prevent it. Therefore, our findings about the role and regulation of ovarian HIF-1alpha signaling provide important clues and directions.

## 4. Hypoxia Inducible Factor-1alpha (HIF-1alpha)

HIF-1alpha is a central regulator of eukaryotic cell and organism metabolism, which is a signaling pathway activated by hypoxia, regulating many gene expressions involved in cell metabolism [74,75,76,77], and playing a critical role in cell survival and normal functions [22,23,24,74,75,76]. Notably, Gregg L. Semenza was awarded the 2019 Nobel Prize owing to his contribution to HIF signaling in cell perception and adaptation to oxygen supply.

### 4.1. The Structure and Function of HIF-1alpha

In 1995, HIF-1alpha cDNA was successfully cloned during the study of erythropoietin (EPO), and subsequent research further elucidated the molecular mechanism by which hypoxia activates HIF-1alpha signaling [19,20,21,22,23,24,25,74,75,76].

**Figure 3:**
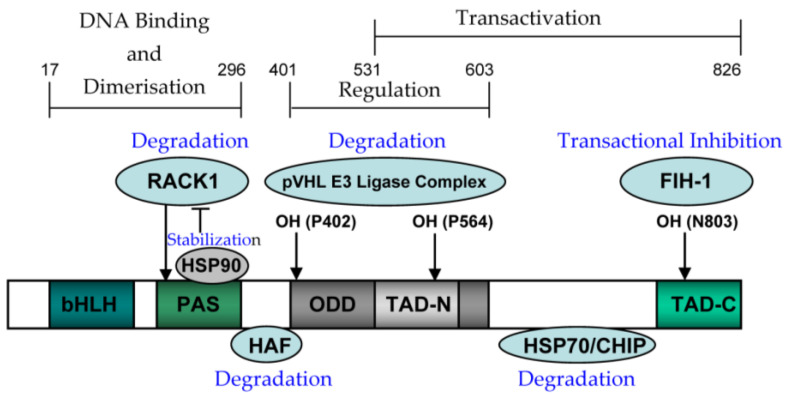
Schematic representation of HIF-1alpha and its functional domains.

Transcription factor HIF-1 is a heterodimer with inducible alpha and constitutive beta subunits, whose amino terminal is composed of bHLH and PAS domains for DNA binding [74]. Its carboxyl terminal is composed of an ODD domain for regulating its stability and a TAD domain for its transcriptional activity (Figure 3) [74]. In addition, the HIF-1alpha terminus has nuclear localization signals, guiding it to the nucleus [23,74,78].

Under hypoxic conditions, HIF-1alpha is transferred to the nucleus and then bonds with HIF-1beta to form a transcriptional activity heterodimer, which binds to the hypoxia response element (HRE) in the target gene promoter, thereby activating transcription and participating in the regulation of various physiological activities, including cell proliferation [79,80,81], metastasis [82,83,84], glycolysis [81,85,86], and angiogenesis [83,87,88].

### 4.2. The Expression and Regulation of HIF-1alpha

HIF-1alpha is expressed in almost all histiocytes, including ovarian granulosa cells [19,20,21,22,23]. HIF-1alpha is the main regulatory factor of cells responses to hypoxic environments by activating its target gene expressions for enhancing tissue oxygen transport or promoting cell metabolism, such as VEGF, EPO, GLUT1, phosphofructose kinase, and lactate dehydrogenase A [89,90,91,92]. It should be emphasized that the selectivity of HIF-1alpha for many genes is highly specific for cell type [89,90,91,92,93]. The difference in HIF-1alpha’s tissue-specific effects on target genes is attributed to the interaction of the HIF-1alpha TAD domain with other transcription cofactors [89,90,91,92,93,94].

**Figure 4:**
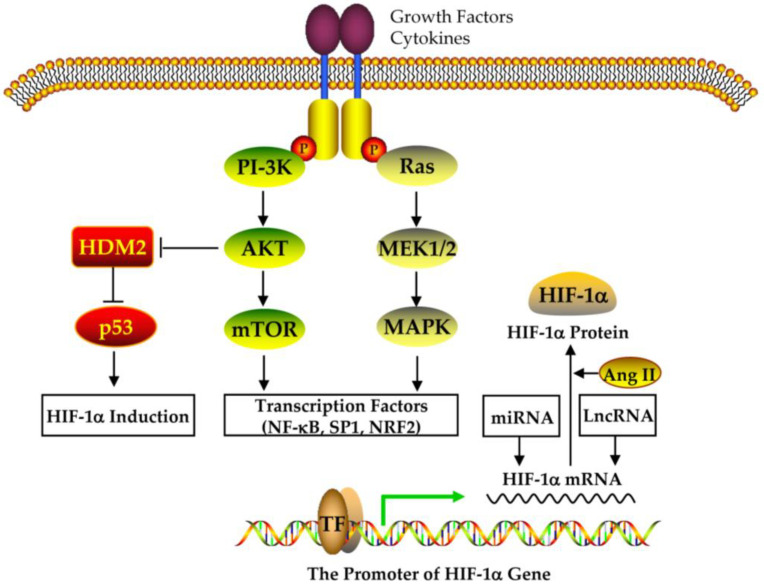
The regulation of HIF-1alpha expression.

**Figure 5:**
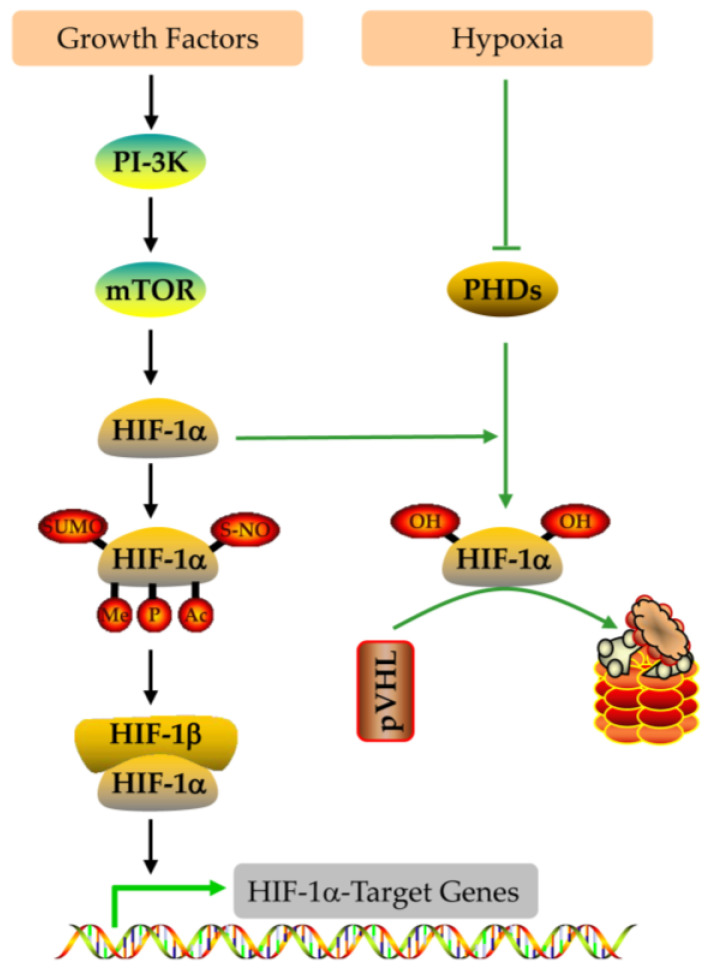
Regulation of HIF-1alpha activity and degradation.

HIF-1alpha expression is regulated at different levels (Figure 4 and Figure 5) [95,96,97,98,99,100,101,102,103,104,105,106]. (1) At the transcriptional level, ROS can induce HIF-1alpha mRNA transcription in an NF-κB-dependent manner [95]. HIF-1alpha transcription also depends on the binding of the specific transcription factor to the SP1 site in their promoter (Figure 4) [96]. (2) At the post-transcriptional level, miRNA-155 targets HIF-1alpha [97], while miRNA 30c-2-3p targets EPAS1 in hypoxia-induced hypoxemia (Figure 4) [98,99,100]. (3) At the translation level, Ang II increases ROS-PI-3K-mediated translation of HIF-1alpha (Figure 4) [101]. (4) At the post-translational level, the E3 ligase complex recruited by VHL is effective against HIF-1alpha hydroxylation, which is the main modification regulating its stability [102]. In addition, HIF-1alpha phosphorylation, acetylation, SUMO acylation, S-nitrosylation, and methylation also affect its stability and activity (Figure 5) [103,104,105,106].

HIF-1alpha activity is affected by many factors, including the accessibility and modification of chromatin DNA, but at least HRE is required in these target gene promoters [107,108,109]. Additionally, HIF-1alpha transcriptional complexes also affect their transcriptional activity, which requires the assembly of HIF-1alpha coactivators and the recruitment of RNA polymerase (Figure 4) [110,111,112].

HIF-1alpha degradation is mainly mediated by the pVHL-mediated ubiquitin proteasome pathway, while hypoxia can block this degradation, leading to HIF-1alpha accumulation [93]. HIF-1alpha ODD domain contains two hydroxylation sites, Pro 402 and Pro 564, which can be hydroxylated by HIF proline hydroxylase [94]. Acetyltransferase ARD1 can also interact with the HIF-1alpha ODD domain and acetylate HIF-1alpha Lys 532 (Figure 5) [113].

Notably, there is a negative feedback regulation mechanism in the body that can prevent HIF-1alpha from continuously activating [19,74,78,94]. For example, activated HIF-1alpha induces PHD2 mRNA expression, which can inhibit HIF-1alpha-dependent gene responses, and this regulation is independent of oxygen concentration [78,94]. Additionally, our previous research found that catalase and ascorbate can block the inhibitory effect of ROS on PHD2 activity [93], demonstrating that antioxidants can regulate HIF-1alpha signaling.

Together, it can be seen that CGA, as an antioxidant, may exert the therapeutic effect of PCOS by inhibiting PHD2 activity and regulating HIF-1alpha-mediated ovarian functions.

## 5. Therapeutic Effect and Regulation of CGA on PCOS

**Figure 6:**
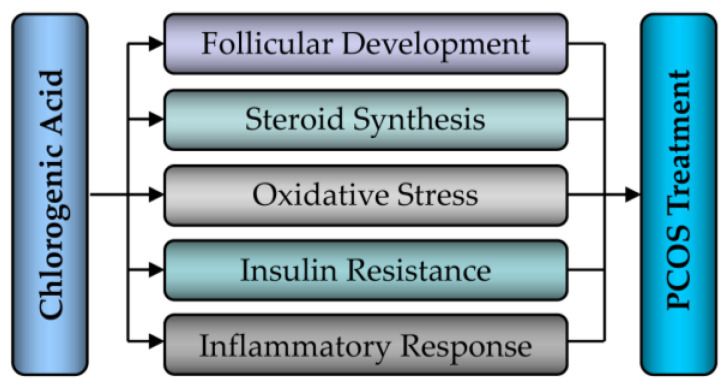
The therapeutic effects of chlorogenic acid (CGA) on PCOS.

Recently, some research on PCOS has found that CGA can not only improve the clinical symptoms of PCOS patients but also the ovarian functions of PCOS rats, indicating the therapeutic effect of CGA on PCOS [10,30,34,35,36,37,45,114]. Based on our previous research on HIF-1alpha signaling during PCOS development and treatment, the present article will systematically elucidate the role and molecular mechanisms of HIF-1alpha signaling during CGA treatment of PCOS from aspects such as ovarian follicle development, steroid hormone synthesis, inflammatory response, oxidative stress, and insulin resistance (Figure 6).

### 5.1. Effect of CGA on Follicular Development in PCOS

Abedpour et al. first discovered that CGA can enhance the in vitro developmental potential of ovarian follicles by reducing oxidative stress and enhancing antioxidant capacity [32], and then they also found that CGA can significantly improve the development of ovarian follicles and the functions of PCOS neuroendocrine in 2022 [30]. In the second year, Shah et al. found that CGA can restore ovarian functions in letrozole-induced PCOS [34]. Intraperitoneal injection of 100 mg/kg CGA improved ovarian structure and resulted in the growth of preantral follicles and the absence of large cysts in the ovarian cortex [34]. In addition, a small amount of corpus luteum was observed in the presence of CGA [37,45]. These research results have shown the therapeutic effect of CGA on PCOS. Therefore, we will elaborate on the molecular mechanism by which CGA improves follicular development in PCOS ovaries in this section based on our previous research.

**Figure 7:**
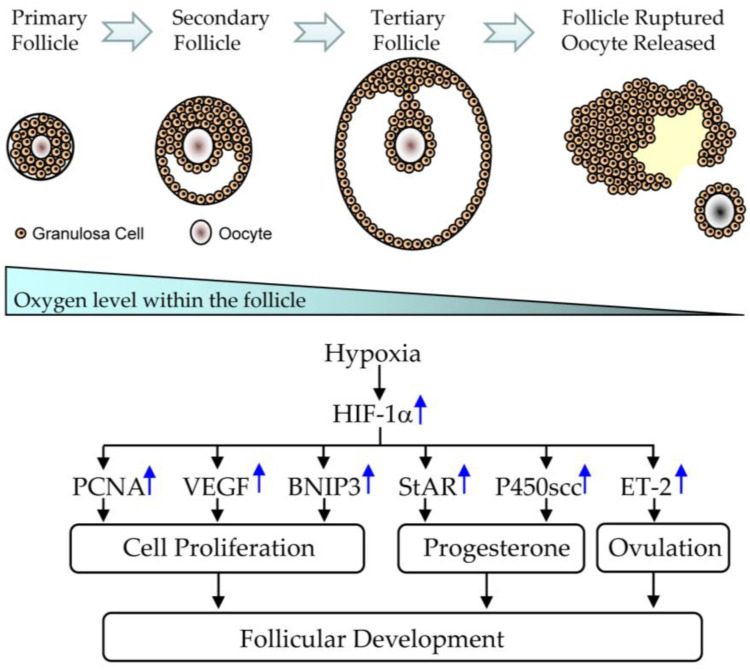
The hypoxic condition and HIF-1alpha-induced genes during the development of ovarian follicles.

During the development of ovarian follicles, the cumulus-oocyte complex is surrounded by the follicular structure, and blood supply is limited to the follicular theca and does not penetrate the basal membrane [19,21,22]. The granulosa cell layer remains avascular until the basal membrane ruptures [19,21]. Therefore, compared to atmospheric oxygen tension, granulosa cells are considered to be in a state of low oxygen tension, or hypoxia [22]. After ovulation, due to bleeding and immature angiogenesis, the ruptured follicle is also considered to be in a hypoxic state [22]. With the expanded blood vessels passing through the basement membrane, a luteal vascular network is established, providing a channel for each luteal cell to enter the capillary [23]. Therefore, all these processes are related to the increased steroidogenic activity that occurs under hypoxic conditions (Figure 7).

HIF-1alpha is an oxygen-regulated transcriptional activator, which is an important factor during hypoxic responses [74]. Under hypoxic conditions, HIF-1alpha becomes stable and then transfers into the nucleus for dimerization with the beta subunit [74,78], which can bind to the HRE of target genes, initiate their transcriptional expressions, and then participate in the regulation of many physiological functions [76,78]. In addition to hypoxia, many inflammatory factors and reproductive hormones can also induce HIF-1alpha expression under normoxic conditions, such as prostaglandins, interferon, or growth factors [74]. More and more evidence suggests that HIF-1alpha participates in the processes of follicular differentiation and ovarian ovulation [22,23,24].

Currently, a large number of studies have shown that HIF-1alpha is mainly expressed in granulosa cells, regulating ovarian functions by the transcription of specific target genes [19,21,22,23]. For example, PMSG not only induces follicle development but also increases HIF-1alpha/PCNA expression, indicating that HIF-1alpha can participate in the regulatory process of follicle development through PCNA-dependent proliferation. In addition, vascular proliferation is accompanied by follicle development. FSH can not only induce VEGF expression in granulosa cells, but this induction can also be blocked by echinomycin, indicating that HIF-1alpha can also participate in the regulatory process of follicle development through VEGF-dependent angiogenesis [22,23,24].

Endothelin-2 is another HIF-1alpha target gene during ovulation [115,116]. Endothelin-2 can induce rapid rupture since it can diffuse to the smooth muscle cells in the outer membrane through the weakened follicle walls [24,116,117]. The contraction of smooth muscle cells leads to follicle contraction, increasing follicle pressure, resulting in rupture at the lowest integrity of the follicular structure [115,116]. In addition to follicle rupture, increased endothelin-2 may promote angiogenesis, cell proliferation, and differentiation [117,118]. Therefore, a decrease in endothelin-2 production in PCOS women may interfere with follicle rupture and subsequent ovulation.

### 5.2. Effect of CGA on Steroid Synthesis in PCOS

**Figure 8:**
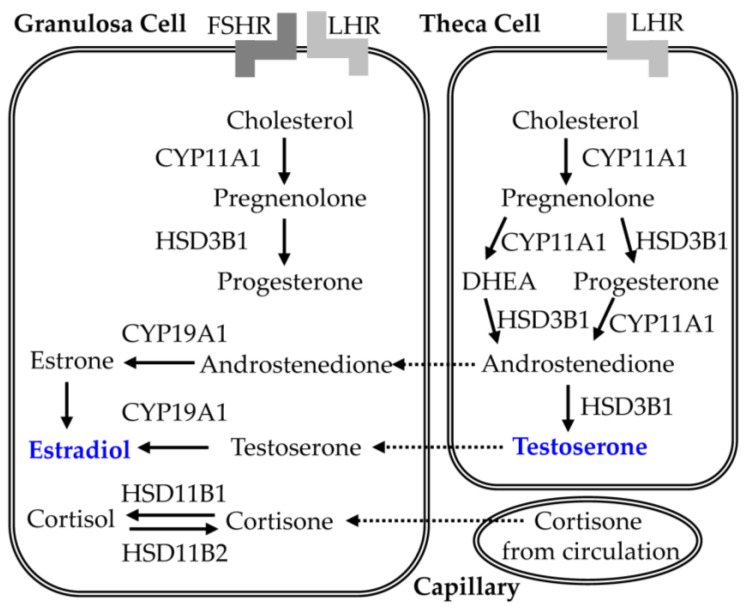
The synthesis of estradiol by two cells and two gonadotropins.

The disorder of GnRH pulse frequency in PCOS is a heterogeneous hormonal imbalance disorder [119,120,121]. In normal ovaries, estrogen is mainly produced by androgen conversion, when LH binds to its receptors on thecal cells of follicles, converting cholesterol into androstenedione and accelerating its secretion into granulosa cells, which convert androstenedione into estrogen through aromatase under the action of FSH. This is the theory of “two cells and two gonadotropins” (Figure 8). Compared with the control, serum progesterone and estrogen concentrations are significantly lower in PCOS patients, while serum androstenedione concentrations are significantly higher [8,58], which may be caused by the abnormal functions of PCOS ovaries.

HIF-1alpha participates in the development of ovarian follicles by regulating the transcription of steroidogenic genes such as StAR, HSD3B, and CYP19A1 [122,123,124]. StAR is one of the key proteins during progesterone synthesis, which can transfer cholesterol through the mitochondrial membrane and is a rate limiting step during steroid synthesis [122,123]. In addition, HSD3B catalyzes the conversion of pregnenolone to progesterone, and aromatase CYP19A1 converts androgen into estrogen, which is an essential hormone for females. Interestingly, the regulation of HIF-1alpha on these three steroidogenic genes is dynamic and tissue-dependent [122]. HIF-1alpha can induce StAR expression in mouse KK1 cells but inhibit StAR expression in mouse Leydig cells [123]. HIF-1alpha can induce HSD3B expression in Leydig cells but inhibit HSD3B expression in canine luteal cells. Similarly, HIF-1alpha can induce CYP19A1 expression in breast adipose stromal cells but inhibit CYP19A1 expression in cortical cells H295R that produce adrenal steroids. In ovarian granulosa cells, hypoxia can induce the expression of STAR and HSD3B through increasing HIF-1alpha activity, leading to an increase in progesterone synthesis [124]. Further research has found that StAR is also a HIF-1alpha target, which can directly bind to the promoter of StAR in granulosa cells under hypoxic conditions, participate in the regulation of StAR transcription, and then increase the steroidogenic capacity of granulosa cells [122,123]. CYP19A1 is a key gene for the production of estradiol in granulosa cells and a downstream target gene of HIF-1alpha [122,124]. In FSH-treated granulosa cells, HIF-1alpha can directly bind to the CYP19A1 promoter and then regulate transcription, leading to an increase in estradiol production in a dose-dependent manner [122].

Recently, research on CGA has found that, compared with the control of PCOS, CGA treatment significantly reduces the concentrations of serum LH and testosterone and significantly increases the concentrations of FSH and progesterone [30,34]. Antioxidants can activate the expression of aromatase, thereby improving follicular development [34,114]. On the one hand, CGA stimulates the expression of cytochrome P450 aromatase in granulosa cells and thecal cells of follicles. On the other hand, CGA reduces serum LH levels in PCOS mice by inhibiting nitric oxide synthase activity and balances the LH/FSH ratio, promoting the development of ovarian follicles [32,35,45].

Together, CGA can regulate the neuroendocrine system of PCOS patients through HIF-1alpha-mediated synthesis of steroid hormones and thus exert its therapeutic effect on PCOS.

### 5.3. Effect of CGA on Inflammatory Response in PCOS

**Figure 9:**
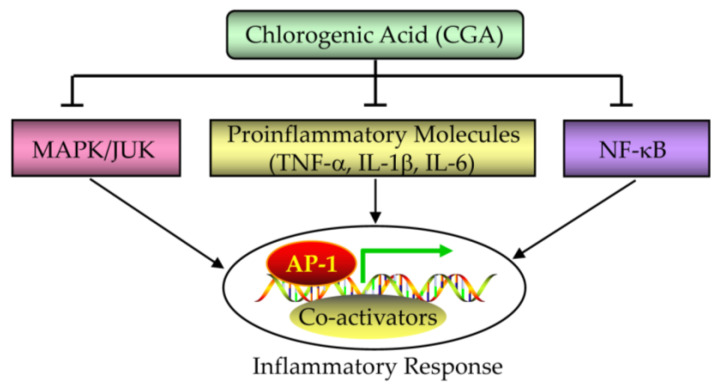
Effect of CGA on the inflammatory response.

PCOS patients often have chronic inflammatory reactions, while CGA has anti-inflammatory effects. Therefore, CGA treatment may eliminate PCOS inflammation (Figure 9) [32,33,34,35,125,126,127].

Inflammation is a defensive response regulated by multiple signaling pathways, while excessive inflammation can damage healthy tissues, leading to organ dysfunction [128,129,130]. Inflammation is always triggered and is involved in the progression of many diseases [128]. Interestingly, HIF-1alpha activation can promote barrier function enhancement [131] and epithelial mesenchymal transition [132]. And artificial activation of HIF-1alpha can improve the prognosis, as demonstrated by the therapeutic effects of HIF proline hydroxylase inhibitors FG-4497 and AKB-4924 in a trinitrobenzene sulfonic acid-induced mouse ulcerative colitis model [133,134,135].

The inflammatory response is triggered and initiated by exogenous stimuli, leading to adaptive changes like the leukocyte exuding and the macrophage activating. Additionally, multiple cytokines and chemical mediators are secreted, and many immunoglobulins are produced during the inflammatory process [136,137]. TNF-α is a major inflammatory initiating factor that induces other cytokines that initiate polymorphonuclear leukocytes and also up-regulates adhesion molecules [138].

The NF-κB signaling pathway can be activated by various inflammatory stimuli and then translocated for increasing specific gene expressions, leading to immune regulation and cell survival [129,138,139]. NF-κB also induces iNOS expression, resulting in the production of the pro-inflammatory mediator NO, which contributes to inflammatory pathogenesis [130,140]. Therefore, more attention was drawn to the characterization of new substances adjusting the excessive production of NF-κB and pro-inflammatory mediators [141].

The immunomodulatory effect of herbal medicine has recently attracted the attention of researchers based on plant immune modulators used to prevent the progression of inflammatory diseases [142]. CGA may be a promising agonist for treating inflammatory diseases due to its powerful immune regulatory effect. Shi et al. found that supplementing CGA can regulate liver fibrosis and inflammation by inhibiting NF-κB activation, serum TNF-α levels, and IL-1β expression [143,144]. CGA can inhibit isoproterenol-induced cardiomyocyte hypertrophy by reducing NF-κB activation [145]. Similarly, CGA can inhibit the migration of neutrophil cells during inflammation [144,146].

### 5.4. Effect of CGA on Oxidative Stress in PCOS

CGA contains orthophenolic hydroxyl groups, which are easily oxidized, resulting in its strong ability to capture and eliminate reactive oxygen species (ROS) and other free radicals [147]. CGA can also block the production of ROS by inhibiting oxidase activity [144,147]. At the same time, it has indirect antioxidant effects by protecting endogenous antioxidant enzymes [148]. Therefore, CGA is widely used because of its good antioxidant activity.

Currently, CGA has been used to improve the treatment of various diseases. For example, CGA can effectively reduce blood and liver lipid accumulation by enhancing its antioxidant activity and regulating lipid metabolism in hyperlipidemic mice [149,150]. CGA can also activate Nrf2/HO-1 and block NF-κB signaling, preventing diabetic nephropathy [151]. CGA can improve NO bioavailability by inhibiting NADPH oxidase activity produced by ROS and the production of superoxide anion, thereby improving vasodilation and endothelial dysfunction in SHR rats [152]. Tsai et al. found that CGA can increase SIRT1 and PGC-1 activity to improve mitochondrial function in HUVECs, thereby reducing Ox-LDL-induced apoptosis [153]. Additionally, CGA can reduce ROS production by increasing intracellular storage of glutathione in human liver cancer cells, thereby limiting oxidative stress-induced apoptosis [30].

Importantly, CGA can effectively improve the structure of ovarian cells and reduce the number of follicular cysts in PCOS ovaries, playing a therapeutic role in PCOS [34,35]. CGA can limit the apoptosis associated with oxidative stress by reducing ROS production and increasing intracellular glutathione levels [154]. For example, CGA, as an effective antioxidant, can enhance the developmental ability of pig oocytes and protect them from DNA breakage caused by H_2_O_2_ exposure [125]. Glutathione is an important regulator of DNA repair activity, so CGA can play an important role in preventing DNA breakage by increasing glutathione levels to combat oxidative stress [155,156,157]. In addition, CGA can provide hydrogen atoms to eliminate hydroxyl radicals, thus protecting DNA from H_2_O_2_-induced damage [158,159]. CGA can also increase antioxidant system contents, including SOD, catalase, and glutathione [160], which may be one of the mechanisms of CGA in treating PCOS.

### 5.5. Effect of CGA on Insulin Resistance in PCOS

PCOS women typically have insulin resistance, which is related to high androgen levels and anovulation, indicating the important role of insulin resistance in the pathophysiology of PCOS [161,162,163,164,165]. In PCOS ovaries, selective insulin resistance mainly affects PI-3K-mediated insulin metabolism rather than MAPK-mediated mitogenic effects [166,167,168]. Defects in the PI-3K signaling pathway inhibit its downstream signaling in PCOS ovaries, thereby affecting the uptake of insulin on glucose [21].

**Figure 10:**
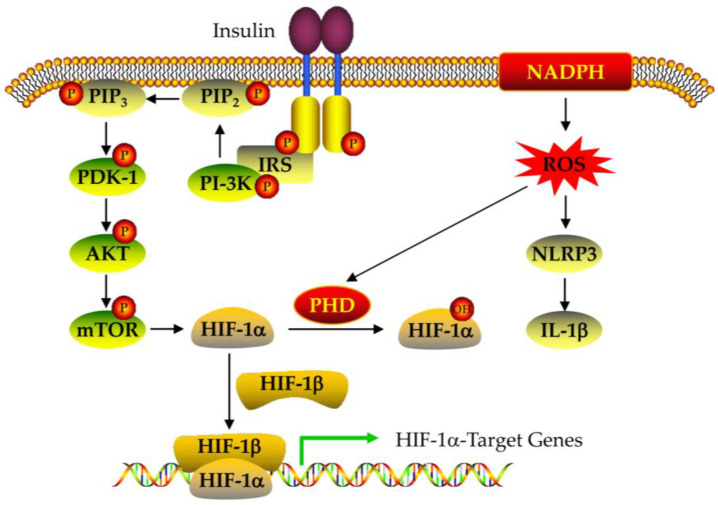
Defects in the insulin signaling pathway in PCOS ovaries.

Insulin activates the insulin receptor substrate (IRS) by binding to its receptor. The phosphorylated tyrosine residue interacts with PI-3K, leading to the phosphorylation of PIP2, producing the second messenger PIP3, which then activates PDK-1 (Figure 10) [169]. After PI-3K activation, signal transduction can propagate to various substrates, such as mTOR [170]. In addition, when mTOR is inhibited in preovulatory follicles, hCG-induced ovulation is also affected [171]. Wang et al. reported that PI-3K p85 significantly reduced mRNA and protein levels in PCOS ovaries, while DMBG can rescue PI-3K expression [21].

DMBG is a common insulin sensitizer that can control the blood sugar of type 2 diabetes, alleviate the clinical symptoms of PCOS patients, and reduce LH levels and hyperandrogenism. DMBG can regulate insulin sensitivity and glucose metabolism in the target tissue of PCOS patients and restore ovulation by enhancing glucose uptake, resulting in reduced insulin synthesis and secretion [20,21,28,29].

CGA is a novel insulin sensitizer like DMBG. Therefore, CGA can improve insulin-mediated PI-3K/mTOR signaling defects in PCOS ovaries.

## 6. Clinical Development of HIF Proline Hydroxylase Inhibitors

**Figure 11:**
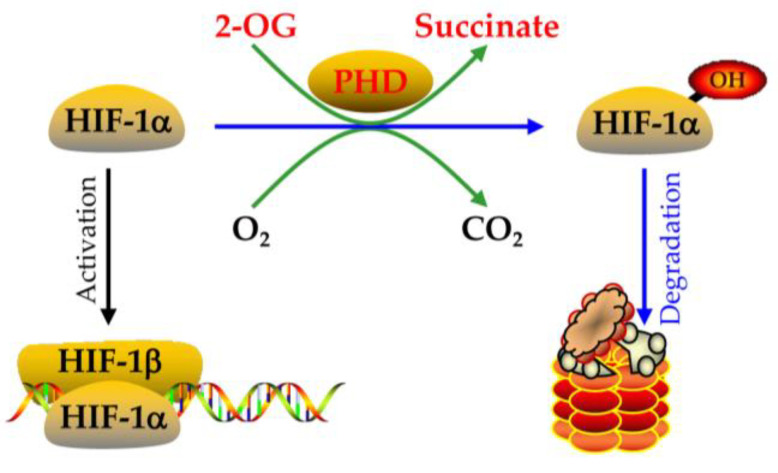
The hydroxylation of HIF-1alpha by 2-oxoglutarate (2-OG)-dependent HIF proline hydroxylase (PHD) and the degradation of HIF-1alpha through the E3 ubiquitin ligase complex.

HIF-1alpha was hydroxylated by 2-oxoglutarate (2-OG)-dependent HIF proline hydroxylases and then degraded through the E3 ubiquitin ligase complex recruited by VHL (Figure 11). HIF proline hydroxylases are called oxygen sensors; their substrates are HIF-1alpha, 2-OG, and O_2_, and their cofactors are Fe^2+^ and ascorbic acid [110,172,173]. In specific cell types or environments, HIF-1alpha activity is regulated by many cellular signals, including the availability of physiological gases other than oxygen (such as NO, CO_2_, and H_2_S), ROS, and HIF hydroxylase cofactors (such as Fe^2+^ and 2-OG).

HIF-1alpha protein can be accumulated by some small-molecule compounds, increase their transcriptional activity, and be independent of oxygen concentration [174,175,176]. But they are nonspecific to HIF proline hydroxylase, and specific to other iron-dependent signaling, leading to excessive toxicity [174,175,176]. For example, Co^2+^, Cu^2+^, and Ni^2+^ salts act as antagonists of Fe^2+^ to inhibit HIF proline hydroxylase activity [177,178]. Iron chelating agents, such as deferoxamine and quercetin, can also inhibit the activity of HIF proline hydroxylases [174,175,176].

Dimethylglycine (DMOG) is a 2-OG antagonist and N-Oxylglycine (NOG) precursor that can inhibit the activity of HIF proline hydroxylases and is mainly used as an HIF-1alpha activator in basic experiments [179]. At present, the molecules entering clinical applications are their derivatives of 2-OG (Table 2), which are specific inhibitors of HIF proline hydroxylase and have negligible inhibitory effects on HDAC and other enzymes [180].

Therefore, considering the therapeutic effect of CGA on PCOS through HIF-1alpha signaling, HIF proline hydroxylase-specific inhibitors alone or in combination are expected to be used for the clinical treatment of PCOS.

## 7. Conclusions and Prospect

Although PCOS is the most common endocrine and metabolic disorder that can lead to female infertility, paying attention to the therapeutic effect of CGA on PCOS will contribute to public health. CGA can reduce ovarian cysts, eliminate oxidative stress, and also be used to improve ovarian functions in PCOS. In addition, CGA may have a therapeutic effect on PCOS through the synergistic effect of decomposing metabolites in the circulatory system. HIF-1alpha, as one of its downstream specific targets, helps us comprehensively and deeply understand the action mechanism of CGA.

Furthermore, CGA is a natural product extracted from fruits, vegetables, and coffee, with an intake equivalent to the daily intake and no safety issues. However, its low bioavailability severely inhibits its clinical potential. In order to improve the bioavailability and tissue distribution of CGA, it is necessary to cleverly modify its structure, optimize a series of delivery systems, and develop targeted formulations to enhance its bioavailability and maintain its important biological activity.

## Figures and Tables

**Table 1:** Diagnostic criteria, clinical phenotype, and treatment focus of PCOS.

Items	Specific Descriptions
Diagnostic Criteria (Rotterdam 2003)	Oligo or anovulation
	Hyperandrogenism
	Polycystic ovaries
Diagnostic Criteria (AE-PCOS Society 2006)	Biochemical and clinical evidence of hyperandrogenism
	Dysfunctional ovaries
	Polycystic ovary morphology
Clinical Phenotype	Hyperandrogenism + Oligo-Anovulation + Polycystic ovaries
	Hyperandrogenism + Oligo-Anovulation
	Hyperandrogenism + Polycystic ovaries
	Oligo-Anovulation + Polycystic ovaries
Treatment Focus	Suppressing and counteracting androgen secretion and action
	Protecting the endometrium and improving menstrual dysfunction
	Improving metabolic status
	Improving ovulatory fertility

**Table 2:** Specific inhibitors of HIF proline hydroxylase available in the clinical field.

Product	Chemical Name	Molecular Formula	Canonical SMILES	Molecular Weight	Molecular Structure
Daprodustat	2-(1,3-dicyclohexyl-2,4,6-trioxohexahydropyrimidine-5-carboxamido)acetic acid	C_19_H_27_N_3_O_6_	O=C(O)CNC(C(C(N1C2CCCCC2)=O)C(N(C1=O)C3CCCCC3)=O)=O	393.43	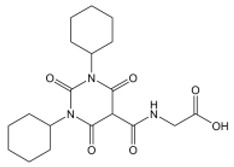
Enarodustat	N-[7-Hydroxy-5-(2-phenylethyl)[1,2,4]triazolo[1,5-a]pyridine-8-carbonyl]glycine	C_17_H_16_N_4_O_4_	O=C(C(C1=NC=NN1C(CCC2=CC=CC=C2)=C3)=C3O)NCC(O)=O	340.33	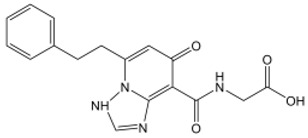
Roxadustat	2-[(4-hydroxy-1-methyl-7-phenoxyisoquinoline-3-carbonyl)amino]acetic acid	C_19_H_16_N_2_O_5_	CC1=NC(=C(C2=C1C=C(C=C2)OC3=CC=CC=C3)O)C(=O)NCC(=O)O	352.34	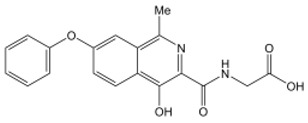
Vadadustat	2-(5-(3-chlorophenyl)-3-hydroxypicolinamido)acetic acid	C_14_H_11_ClN_2_O_4_	OC1=CC(C2=CC=CC(Cl)=C2)=CN=C1C(NCC(O)=O)=O	306.70	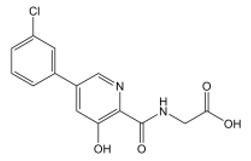
Molidustat	2-(6-morpholinopyrimidin-4-yl)-4-(1H-1,2,3-triazol-1-yl)-1H-pyrazol-3(2H)-one	C_13_H_14_N_8_O_2_	O=C1C(N2C=CN=N2)=CNN1C3=NC=NC(N4CCOCC4)=C3	314.30	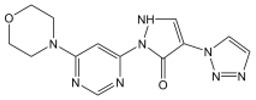

## Data Availability

Not applicable.
